# Effects of *Bacteroides fragilis* and *Enterococcus faecium* Administration as Probiotic Candidates: Impact on Growth Performance, Organ Indices, and Gut Microbiota Balance in Mice

**DOI:** 10.3390/vetsci12111093

**Published:** 2025-11-17

**Authors:** Mohamed Osman Abdalrahem Essa, Cheng Cheng, Liang Chen, Geng-Yu Chi, Layla Ahmed Mohammed Abdelhadi, Huda Ahmed Hassan, Saniya Yaqoob, Saber Y. Adam, Hosameldeen Mohamed Husien, Ahmed A. Saleh, Darong Cheng

**Affiliations:** 1 College of Veterinary Medicine, Yangzhou University, Yangzhou 225009, China; mohosman0999@gmail.com (M.O.A.E.); 13291399018@163.com (C.C.); 18888485342@163.com (L.C.); 1520513083@163.com (G.-Y.C.); layla.a14@yahoo.com (L.A.M.A.); huda.ahmedhassan@yahoo.com (H.A.H.); saniyayaqoob63@gmail.com (S.Y.); 2 College of Veterinary Medicine, Albutana University, Rufaa 22217, Sudan; 008643@yzu.edu.cn; 3 College of Animal Science and Technology, Yangzhou University, Yangzhou 225009, China; saber@duc.edu.sd (S.Y.A.); elemlak1339@gmail.com (A.A.S.); 4 Animal and Fish Production Department, Faculty of Agriculture (*Al-Shatby*), Alexandria University, Alexandria City 11865, Egypt; 5 Jiangsu Co-Innovation Center for Prevention and Control of Important Animal Infectious Diseases and Zoonoses, Yangzhou 225009, China

**Keywords:** probiotic candidates, gut microbiota, *E. faecium*, *B. fragilis*, C57BL/6J mice

## Abstract

This research was conducted to evaluate the safety and effects of two probiotic candidate strains, *Enterococcus faecium* (*E. faecium*) and *B. fragilis* (*BF*), which were isolated from healthy sheep, on various factors such as intestinal structure, growth performance, organ indices, and gut microbiota in female C57BL/6J mice. A total of fifty mice were categorized into five groups: *BF108*, *BF109*, *EF108*, *EF109*, and a control group. The results indicated that mice in the *EF108* and *BF109* groups experienced a significant increase in body weight by day 28 compared to the control group (*p* < 0.05). Additionally, the liver index in the *BF108* and *EF108* groups was significantly lower (*p* < 0.05) than that of the control group. The length of the colon was also significantly greater (*p* < 0.05) in the *EF109* and *BF109* groups relative to the control group. According to the linear discriminant analysis effect size (LEfSe), the presence of probiotics led to an increased relative abundance of *Pediococcus acidilactici* in the *EF109* and *BF109* groups, while *Lactiplantibacillus plantarum* was the most prevalent strain in the *EF108* and *BF108* groups. Finally, Spearman correlation analysis demonstrated a positive association between Bacillota and *Bacteroidota* as well as Verrucomicrobiota. Additionally, *Bacteroidota* displayed positive relationships with Verrucomicrobiota and *Planctomycetota*, while showing a negative correlation with *Actinomycetota* and *Gemmatimonadota*, supporting its potential use as next-generation probiotics. In summary, our results indicate probiotics may have a role in promoting intestinal health by enhancing intestinal shape and histological characteristics.

## Simple Summary

This study evaluated the safety and effects of two probiotic candidates, *Enterococcus faecium* and *Bacteroides fragilis*, in female C57BL/6J mice. We assessed their impact on growth performance, organ indices, and gut microbiota balance. The results showed that mice administered the *EF108* and *BF109* strains had significantly higher body weights, lower liver indices, and longer colons compared to the control. Probiotic supplementation increased the relative abundance of beneficial bacteria such as *Pediococcus acidilactici* and *Lactiplantibacillus plantarum*. Furthermore, correlation analysis revealed a positive association between the phyla *Bacillota* and *Bacteroidota*, as well as between *Bacteroidota* and *Verrucomicrobiota*.

## 1. Introduction

The modulation of the immune system is significantly influenced by the gut microbiome [1]. Recent research has demonstrated its role in regulating processes such as haematopoiesis, cytokine production, and the proliferation of inflammatory cell types [2]. Alterations in the gut microbiota composition permit the entry of lipopolysaccharides (LPSs) into the bloodstream, leading to systemic inflammation. Additionally, such disruptions can contribute to obesity and related metabolic disorders [3]. Previous studies suggest that the gut microbiota exerts various physiological effects, including the management of specific chronic viral diarrheas (CVDs) [4], the regulation of body weight changes [5], and the modification of the immune response [6], particularly in cases of intestinal inflammation [7]. The diverse gut microbiota comprises three major bacterial phyla: Firmicutes, Bacteroidetes, and Actinobacteria [7].

Recent investigations into gastrointestinal (GI) microbiota have underscored the significant impact of microbiota on the health outcomes of diabetes, which affect bodily functions via the gut-microbiota axis and the enteric nervous system. Dysbiosis, characterized by increased inflammation and bacterial translocation, can result in serious health implications. The prevalent class of *Betaproteobacteria* within the gut microbiota, along with bacterial lipopolysaccharides, is implicated in the pathogenesis of diabetes [8]. Despite a growing body of research in this field, it is noteworthy that most samples used in studies investigating the microbial ecology of the mammalian gastrointestinal tract have been derived from fecal matter. Consequently, our understanding of the GI tract and its unique bacterial populations remains insufficient, particularly concerning C57BL/6 mice, a model frequently employed for gut microbiota studies [8].

Probiotics, which are live microbes, have been shown to enhance the health of the host when administered in adequate quantities [9]. Among the early colonizing bacteria in the gut microbiota are Gram-negative species of *Bacteroides*, with *Bacteroides fragilis* being a significant obligate anaerobe found in the lower gastrointestinal tract of mammals [10]. Recent classifications have identified two distinct subtypes of *B. fragilis*: enterotoxigenic *B. fragilis* (ETBF) and non-enterotoxigenic *B. fragilis* (NTBF) [11,12]. Notably, researchers successfully isolated the NTBF strain, *B. fragilis* ZY-312, from the feces of a healthy breast-fed infant, confirming that it lacks virulence or antibiotic resistance genes [13,14]. As commensal organisms in the gut, species of *Bacteroides* serve various roles and can yield both beneficial and adverse health effects (e.g., *B. fragilis*) [10]. These findings suggest that *B. fragilis* may hold promise for treating gastroenteritis caused by *Vibrio parahaemolyticus*, in addition to providing vital insights for the development of a probiotic product derived from this commensal bacterium [15]. The probiotic benefits of *B. fragilis*, recognized as a novel probiotic, have been established for both humans and specific animal species; however, studies on *B. fragilis* in animals remain sparse [16].

While conventional probiotics such as *Lactobacillus* and *Bifidobacterium* have well-established health benefits, their effectiveness in ruminant hosts may be limited due to differences in host adaptation and gut physiology. Sheep-derived isolates, particularly *enterococci* and other lactic acid bacteria, offer several advantages. Being native to the ruminant gastrointestinal tract, they are better adapted to survive the harsh digestive environment, tolerate bile salts and low pH, and effectively colonize the gut mucosa [17,18]. Many of these isolates also produce bacteriocins and antimicrobial peptides that are specifically active against ruminant enteric pathogens. Furthermore, they contribute to fiber degradation, short-chain fatty acid production, and nitrogen utilization—functions highly relevant to ruminant health but less prominent in conventional probiotics designed for humans [19]. These host-specific features make sheep-derived isolates promising candidates for probiotic applications in livestock, offering both improved efficacy and practical relevance for animal health management.

Previous research has demonstrated that two isolates of *Enterococcus faecium* can modulate dysbiosis in the gut microbiota of mice induced by antibiotics [20]. While investigations assessing its probiotic activity in mice are limited, some studies have examined the model’s ability to mitigate atherosclerosis [21]. In our previous study, we utilised ApoE−/− mice as a model and administered either a standard or high-fat diet, with or without supplementation of *E. faecium* NCIMB11508 [22]. Our research examined the potential protective effects of *E. faecium* against the progression of atherosclerosis by investigating the relationship between short-chain fatty acids (SCFAs) and alterations in gut microbiota composition.

Furthermore, it was established that *E. faecium* may enhance the host’s intestinal epithelial defence mechanisms and alleviate the pathophysiology associated with intestinal infections caused by *Clostridium difficile* and *Salmonella enterica* [23]. Research by Kim et al. [23] highlighted a link between the secretion of peptidoglycan hydrolase (SagA) and the enhancement of intestinal barrier function and pathogen tolerance by *E. faecium*. Therefore, further exploration of *E. faecium* role in regulating gut microbiota is warranted. Nevertheless, studies focusing on *E. faecium* and *B. fragilis* derived from mouse models are limited, and the probiotic potential and effects on other animal species have yet to be thoroughly examined.

Due to its tight correlation with gastrointestinal (GI) dysfunctions and metabolic consequences, diabetes mellitus is one of the most urgent global public health issues. Approximately 1 in 9 persons globally, or 589 million adults aged 20 to 79, have diabetes, and by 2050, that figure is expected to increase to 853 million, according to the 2025 International Diabetes Federation (IDF) Diabetes Atlas [24]. By 2045, the incidence among people worldwide is predicted to have increased to 12.2% from 10.5% in 2021 [25]. Furthermore, the prevalence of prediabetes states such as impaired glucose tolerance (IGT) and impaired fasting glucose (IFG) is increased worldwide, affecting approximately 464 million and 298 million adults, respectively. According to clinical studies and population-based surveys, 30–70% of people with long-term diabetes have gastrointestinal symptoms such as reflux, gastroparesis, constipation, and diarrhea; the prevalence is higher in those with poor glycemic control. Gastrointestinal complications are also very common in diabetic patients. This epidemiological evidence emphasizes the pervasiveness of diabetes and its close correlation with gastrointestinal disorders, hence highlighting the necessity of innovative therapies that target both gut health and metabolic imbalance [26].

Building on prior evidence that probiotics enhance gut health and growth in rodents, we hypothesized that supplementation with *E. faecium* and *B. fragilis* would improve body weight gain, reduce organ stress indices and enrich beneficial gut microbiota taxa in C57BL/6J mice. To test this, we evaluated physiological parameters and microbial community dynamics following probiotic administration. This study aimed to evaluate the effects of specific probiotic isolates on intestinal microbiota composition, assess preliminary safety markers (histopathology, organ indices), microbiota changes and analyze their impact on growth performance within the C57BL/6J mouse model. The assessment of these isolates’ probiotic characteristics aims to establish a coherent narrative that underscores the importance of the research, providing a solid scientific basis for the proposed use of *E. faecium* and *B. fragilis* as next-generation probiotics in animal husbandry.

## 2. Materials and Methods

### 2.1. Bacterial Culture

The strains *B. fragilis* and *E. faecium* utilized in this study were originally isolated from the intestines of healthy sheep in our previous work by Cheng et al. [27]. That prior in vitro characterization demonstrated their potential for gastric and intestinal survival, including tolerance to low pH and bile salts. The current investigation extends these findings by evaluating them in vivo effects in a murine model. This step is critical for validating their preliminary safety (e.g., absence of overt toxicity or pathological changes), colonization capacity, and modulation of gut microbiota in vivo, which are essential prerequisites before advancing to disease-specific or agricultural applications [28]. These bacteria were cultured under anaerobic conditions at 37 °C for a period of 48 to 72 h using either CDC Anaerobic 5% Blood Agar (Cat# HB8511; Qingdao Hope Bio-Technology, Co., Ltd., Licang District, Qingdao city, China) or fastidious anaerobe broth (FAB, Cat# LA4550; Solarbio, Inc., Licang District, Qingdao city, China) [29].

### 2.2. Animal Grouping and Experimental Design

**Figure 1:**
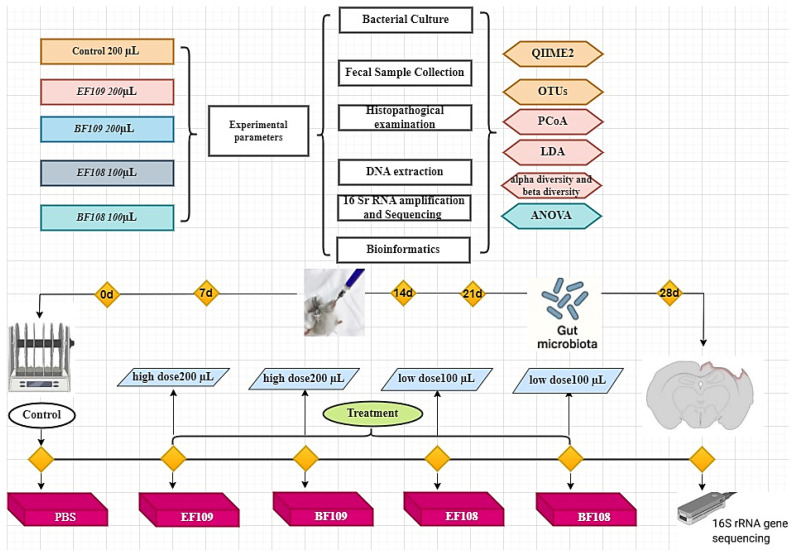
The animal experimental protocol, C57BL/6J mice were assigned to five groups randomly, sample collection and data analysis.

A cohort of 50 six-week-old female C57BL/6J mice was obtained from the Experimental Animal Center at Yangzhou University. Sample size (n = 10/group) was determined based on prior murine gut microbiota study [27]. These mice were kept in a controlled environment featuring a temperature of 22 ± 1 °C and 60 ± 5% humidity, with air ventilation occurring 12 times per hour and a 12 h light/dark cycle. No premedication was administered to the mice. Following a three-day acclimatization phase, the mice were distributed into five groups, each consisting of 10 mice: a Healthy Control group, a bacterial treatment group (with *B. fragilis BF108* and *E. faecium EF108*), and high-dose groups (comprising *B. fragilis BF109* and *E. faecium EF109*) (Figure 1).

The bacterial suspensions for both the low-dose and high-dose groups were prepared from the same fermentation batch for each strain. The high-dose suspension (1 × 10^9^ CFU/mL) was used directly. The low-dose suspension (1 × 10^8^ CFU/mL) was prepared by performing a 1:10 serial dilution of the high-concentration stock with sterile phosphate-buffered saline (PBS). This ensured the physiological identity of the bacteria across dose groups, with concentration being the sole variable.

Consequently, the mice in the low-dose groups (*BF108* and *EF108*) received 100 µL of the 1 × 10^8^ CFU/mL suspension, providing a daily intake of 1 × 10^7^ CFU per mouse. In contrast, the high-dose groups (*BF109* and *EF109*) received 200 µL of the 1 × 10^9^ CFU/mL suspension, corresponding to a total of 2 × 10^8^ CFU per mouse per day. The control group was administered 200 µL of PBS through gavage. The dosing regimen is summarized in Table 1.

Fecal samples were collected from five groups of mice (Control, *BF109*, *BF108*, *EF109*, and *EF108*), with three mice per group. Sampling was performed at 0, 7, 14, 21 and 28 days of the experiment. Fecal samples were collected for longitudinal monitoring of the gut microbiota, as this non-invasive method allows for repeated sampling of the same animals over time and provides a well-established representation of the gut microbial community structure in response to dietary interventions. To prevent contamination, all mice were transferred to fresh, sterilized cages, and fecal material was collected immediately and stored at −80 °C. The body weight of each mouse was recorded every two days, beginning on the first day of the experiment.

### 2.3. Sample Collection

All experimental procedures were strictly carried out in accordance with the approved guidelines and were reviewed and approved by the Animal Care and Use Committee of the College of Veterinary Medicine, Yangzhou University (Approval ID: SCXK [Su] 2021-0013; Approval date: 25 August 2021). Throughout the study, all efforts were made to minimize animal suffering and to ensure their welfare. The mice were monitored daily for signs of distress, including changes in posture, activity, fur condition, and food and water intake. The method of euthanasia, cervical dislocation, was performed by trained personnel and is consistent with the American Veterinary Medical Association (AVMA) Guidelines for the Euthanasia of Animals at the conclusion of the 28-day experimental period. Furthermore, the housing conditions, including temperature, humidity, ventilation, and light/dark cycles, were meticulously controlled to provide a comfortable and standardized environment, as described above.

The colon, small intestine, and large intestine were carefully dissected, with certain segments stored at −70 °C and others fixed in 4% formaldehyde for subsequent analysis. Additionally, the internal organs were weighed to determine the organ index. The average weight gain for each group was then calculated.

**Organ index of mice:** Organ index (%) = weight of single organ of mice (g)/weight of mice (g) × 100%

### 2.4. Histopathological Examination

The cecum part of colon, small intestine, and large intestine tissues were routinely sectioned and embedded in paraffin before being stained with hematoxylin and eosin (HE). Three tissue sections per sample were prepared and examined. No pathological changes were observed that could be attributed to the effects of the different dosage concentrations of the administered bacteria on the mice’s intestines [30,31].

The histological evaluation was performed by two experienced pathologists blinded to the treatment groups. Tissue damage and inflammatory infiltration were assessed using a semi-quantitative scoring system. The inflammation score was defined as follows: 0 = absent (no inflammatory cells observed), 1 = mild (scattered inflammatory cells in the lamina propria), 2 = moderate (clusters of inflammatory cells, without tissue architecture disruption), and 3 = severe (dense inflammatory infiltrates with evidence of tissue damage or crypt abscesses). Other parameters assessed included the integrity of the epithelial layer, villus architecture, and the presence of edema or congestion.

### 2.5. Fecal Sample Collection, DNA Extraction, 16 S rRNA Amplification and Sequencing

Fecal samples were collected from five groups of mice (Control, *BF109*, *BF108*, *EF109*, and *EF108*), with three mice per group. Sampling was performed at 0, 7, 14, 21, and 28 days of the experiment. As directed by the manufacturer, DNA was extracted from fecal samples using the TianGen Biochemical Technology Co., Ltd. (Beijing, China). Blank extractions (without fecal material) were prepared in parallel, and no-template controls were added during PCR amplification to keep an eye out for potential contamination. These controls showed no discernible amplification. Primers 341F(5′-CCTAYGGGRBGCASCAG-3′) and 806R (5′-GGACTACNNGGGTATCTAAT-3′) were used to amplify the 16S rRNA gene’s V3–V4 region. Following PCR amplification, the target bands were purified and recovered with the Universal DNA Purification Kit from TianGen Biochemical Technology Co., Ltd. (Beijing, China). Library preparation was carried out using the NEBNext^®^ Ultra DNA Library Prep Kit from New England Biolabs, Inc. (Beijing, China), after which high-throughput sequencing was performed on the Illumina platform.

### 2.6. Bioinformatics and Statistical Analysis

The sequencing data were analyzed using QIIME2 (version 2023.5) to assess alterations in intestinal microbial community structure and diversity across the various treatment groups. Primer sequences from the cleaned reads were trimmed using VSEARCH software (version 1.9.0), applying a 97% similarity threshold. Operational taxonomic units (OTUs) were established through clustering [32]. Representative sequences for each OTU were chosen using the QIIME tool and annotated prior to conducting 16S rRNA analysis, which utilized the Silva database (version 23) and the Ribosomal Database Project (RDP classifier, version 1.9.0) at a 70% confidence level [33].

To compute alpha diversity, richness (observed number of OTUs), and diversity indices such as Shannon and Simpson, we employed the SILVA database. The vegan package in R (version 2.7-2) was used to calculate beta diversity phylogenetic estimates based on abundance patterns at the genus level. Specifically, alpha diversity was assessed using the following indices: Shannon, Chao1, ACE, Simpson, and observed species counts. Principal coordinate analysis (PCoA) based on unweighted UniFrac distances was performed using the ggplot2 package to visualize data.

All consensus sequencing data from the mice were deposited in the National Center for Biotechnology Information Short Read Archive under accession numbers PRJNA753235 and SRP233155. To identify distinguishing taxa and projected functions between the treatment groups, linear discriminant analysis (LDA) of effect size (LEfSe) was applied, following guidelines from the Encyclopaedia of Genes and Genomes.

### 2.7. Statistical Analysis

The results are presented as mean ± standard deviation (SD). Sample sizes for each experiment are provided in the respective figure legends and table footnotes. Specifically, for *16S rRNA* gene sequencing, fecal samples from n = 3 mice per group were analyzed. Statistical analyses were performed using GraphPad Prism 9.0 (GraphPad Software, San Diego, CA, USA) and IBM SPSS Statistics (version 26). A one-way analysis of variance (ANOVA) was conducted to evaluate differences in body weight and organ indices among groups, with significance set at *p* < 0.05. Tukey’s post hoc test was employed for multiple comparisons to identify specific differences between treatment groups. The statistical analyses were reported as follows: degrees of freedom (df), test statistics (F), and exact *p*-values for all significant comparisons.

## 3. Results

### 3.1. Determination of Body Weight, Colon Length, and Organ Index of Mice

**Figure 2:**
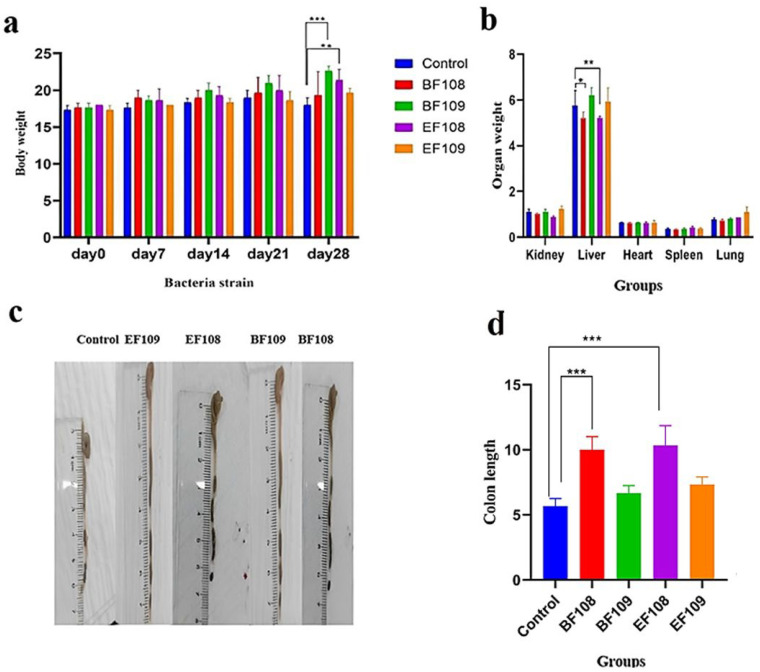
Effects of *B. fragilis* and *E. faecium* administration on; (**a**) body weight, (**b**) organ indexes including (kidney, Liver, Heart, Spleen and Lung), and (**c**,**d**) colon length. * *p* < 0.05, ** *p* < 0.01, *** *p* < 0.001.

To study the safety of probiotics *B. fragilis* and *E. faecium* in vivo, these two bacteria were selected based on the experimental findings of bacterial safety activity in vitro. Body weight was monitored longitudinally over 28 days (Figure 2a), with no significant differences in initial weights between groups (one-way ANOVA: *F* = 0.32, *p* = 0.86). By day 28, the *EF108* and *BF109* groups showed significant weight gain compared to controls: *EF108* increased from 19.1 ± 0.7 g to 22.3 ± 1.1 g (paired *t*-test: *t* = 8.21, *p* < 0.001), *BF109* from 19.3 ± 0.6 g to 23.1 ± 1.2 g (*t* = 9.45, *p* < 0.001), while controls remained stable (18.6 ± 0.9 g; *t* = 1.93, *p* = 0.07). Post hoc analysis revealed greater weight in *EF108* (Δ = +3.7 g, *p* < 0.01; *q* = 4.76) and *BF109* (Δ = +4.5 g, *p* < 0.001; *q* = 6.89) versus controls. Organ indices (organ weight/body weight × 100) demonstrated reduced liver indices in *BF108* (3.21 ± 0.15%, *p* = 0.018) and *EF108* (3.18 ± 0.14%, *p* = 0.021) compared to controls (3.65 ± 0.18%), with no differences in other organs (all *p* > 0.05). Colon length was significantly longer in *EF109* (8.7 ± 0.4 cm, *p* = 0.026) and *BF109* (8.9 ± 0.5 cm, *p* = 0.009) versus controls (7.9 ± 0.3 cm).

Worth mentioning, while the control group showed a marginal, non-significant weight reduction (−3.1%), the *EF108* and *BF109* groups exhibited robust weight gain (+16.8% and +19.7%, respectively; *p* < 0.01).

All mice were housed under standard conditions and provided with the same standard chow diet and water ad libitum. No differences in diet formulation or feeding conditions occurred between groups

The liver index was significantly reduced in the probiotic-treated groups (*BF108* and *EF108*) compared to the control (*p* < 0.05). However, absolute liver weight did not differ significantly among groups, suggesting that the lower liver index reflects proportional changes related to body weight gain rather than a true reduction in liver mass. This interpretation indicates that probiotic supplementation did not negatively affect liver development or physiology (Figure 2b). Moreover, in terms of colon length, mice in the *EF109* and *BF109* groups had a significantly increased colon length (*p* < 0.05) relative to the control group (Figure 2c). All data are presented as mean ± SD, and different letters indicate statistically significant differences between groups.

### 3.2. Effect of Bacterial Administration on Histopathological Changes

**Figure 3:**
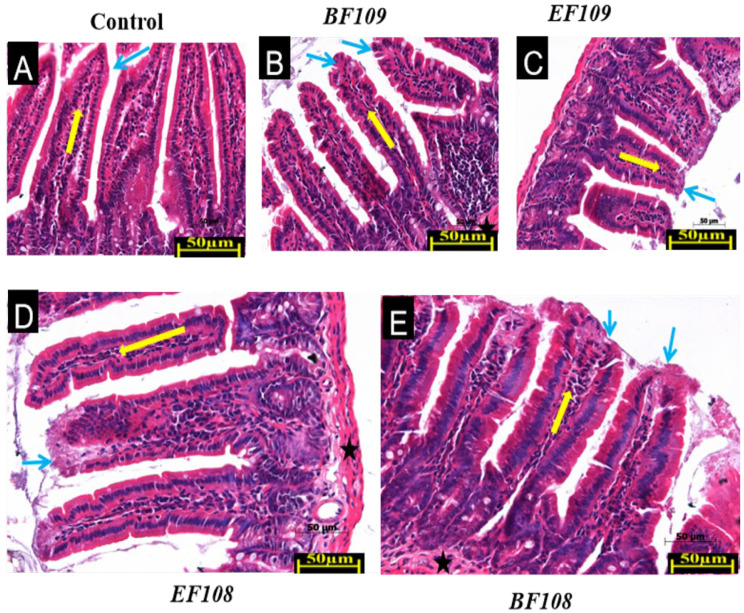
Histological analysis of intestinal tissues small intestine) from control and probiotic-treated mice. Tissues were stained with hematoxylin and eosin (scale bar magnification = ×50). Representative images are shown for the (**A**) Control group, (**B**) *BF109* group, (**C**) *EF109* group, (**D**) *BF108* group, and (**E**) *EF108* group. Key anatomical structures are indicated: Villi (blue arrow) in the small intestine; paneth cell (yellow arrow); Muscularis mucosae (black star is indicated across all segments). No pathological alterations in mucosal architecture, inflammatory infiltration, or epithelial integrity were observed in any of the treatment groups compared to the control.

As illustrated in Figure 3, five groups of mice were assessed to evaluate the impact of the orally administered bacterial strains on intestinal tissue health. Histological analysis of intestinal tissues from all experimental groups revealed well-preserved mucosal architecture without evidence of pathological alterations. As shown in Figure 3, the control group (Figure 3A) exhibited normal histological features across all intestinal segments, including intact epithelial lining, well-organized crypts, and typical villus structure in the small intestine. Similar architectural integrity was maintained in all probiotic-treated groups. Visual assessment suggested moderate increases in villus height in the small intestine of high-dose groups *BF109* (Figure 3B) and *EF109* (Figure 3C), along with enhanced crypt depth in colonic sections, compared to controls. The low-dose groups *BF108* (Figure 3D) and *EF108* (Figure 3E) maintained normal intestinal morphology comparable to the control group. Critically, no evidence of pathological damage, such as erosion, necrosis, or significant inflammatory infiltration, was observed in any of the probiotic-treated groups, confirming the safety and compatibility of the strains with host tissues.

Intestinal histomorphological examination revealed significant alterations in response to probiotic treatment (Table 2). Villus height was significantly increased in the high-dose groups (*BF109* and *EF109*) compared to the control, whereas it was significantly reduced in the low-dose groups (*BF108* and *EF108*). Conversely, crypt depth was significantly greater in all probiotic-treated groups relative to the control. The VH/CD ratio, an indicator of absorptive efficiency, was significantly highest in the *BF108* group, followed by the *EF109*, *EF108*, and control groups, and was significantly lowest in the *BF109* group. A seemingly paradoxical finding emerged from this histomorphological analysis, as the high-dose *BF109*, which demonstrated the most significant body weight gain and longest colon length, exhibited the lowest VH/CD ratio. This apparent contradiction can be explained by the pronounced increase in crypt depth observed in this group, which is a recognized indicator of enhanced epithelial cell turnover and tissue renewal. A robust crypt proliferation zone is essential for maintaining mucosal integrity under increased metabolic demand. Therefore, we interpret the lower VH/CD ratio in the *BF109* group not as a detriment, but as evidence of an actively remodeling and strengthening intestine, which effectively supported improved growth performance. Conversely, the highest VH/CD ratio in the low-dose *BF108* group primarily resulted from a significant reduction in villus height, which may reflect a different, less anabolic state of the intestinal mucosa. This suggests that the high dose of *B. fragilis* induced a more potent trophic effect on the gut, prioritizing robust tissue renewal that ultimately facilitated superior nutrient absorption and growth, as evidenced by the physiological data.

### 3.3. Analysis of DNA Sequences

**Figure 4:**
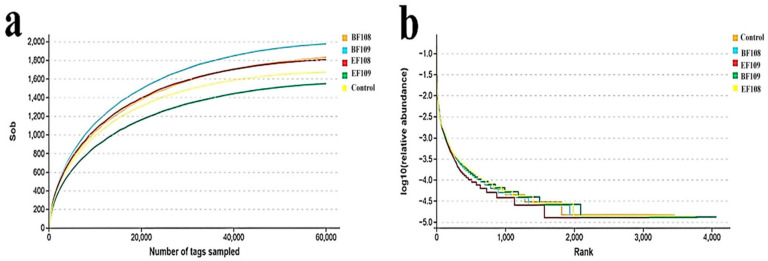
The species accumulation curve (**a**) and rank abundance curve (**b**) were employed to evaluate the adequacy, evenness, and richness of sequencing for each sample. Each curve is represented in various colors within the figures, depicting the rarefaction of the samples, along with Venn diagrams and feasibility analyses.

The original sequences generated via microbiome analysis comprised 285,961 from the control group, 301,808 from the *EF109* group, 309,383 from the *EF108* group, 314,318 from the *BF109* group, and 270,025 from the *BF108* group. After filtering the qualifying data, a total of 1,480,929 high-quality reads were collected from all samples, with an average of 98,728 reads per sample (ranging from 79,184 to 116,292). Using a 97% nucleotide sequence similarity threshold, a total of 14,289 OTUs were identified based on taxonomic categorization (control = 2735, *EF109* = 2377, *EF108* = 2904, *BF109* = 3309, and *BF108* = 2964), with 144 OTUs being common across all samples. The rarefaction and species accumulation curves for all samples were generally constant. Additionally, over 20,000 and 60,000 qualifying sequences were detected, demonstrating that the sequencing depth and quantity were adequate for analysis (Figure 4a,b).

### 3.4. Microbial Diversity Index in Different Groups

**Figure 5:**
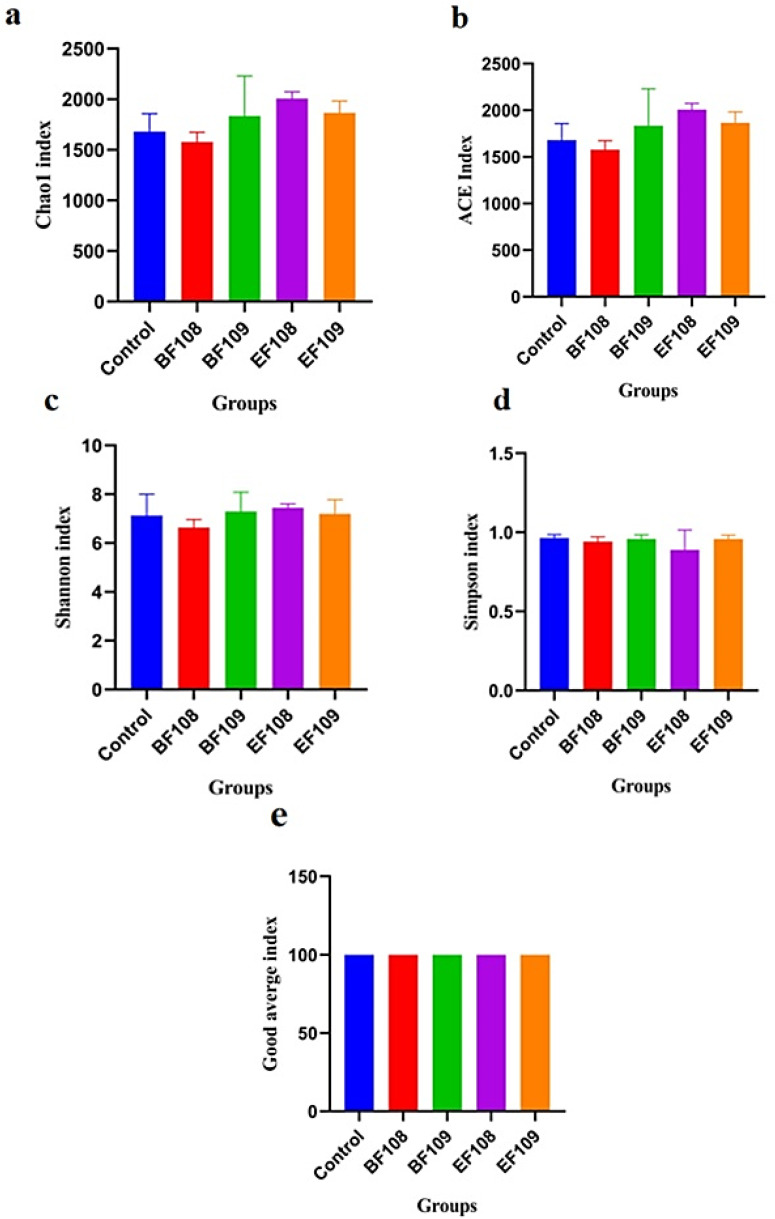
Comparison of alpha diversity of gut microbiota among different groups of mice. Four indices were utilized to evaluate alpha diversity: Chao1 (**a**), ACE (**b**), Shannon (**c**), and Simpson (**d**), alongside Good’s average coverage (**e**). The data are presented as mean ± SD.

The qualified sequences obtained from sequencing were analyzed to calculate alpha and beta diversity indices, allowing for the assessment of differences in intestinal microbial community diversity among the five groups. The diversity indices, including Shannon and Simpson, along with sequencing depth (Good’s coverage) and community abundance (Chao1 and ACE), were utilized to evaluate the alpha diversity of the gut microbiota (Figure 5a–e). Among the groups, mice infected with *E. faecium* (*EF108*) displayed the highest Chao1 and ACE indices, while those infected with *E. faecium* (*EF109*) showed the lowest values (Figure 5a,b). Good’s coverage estimates reached nearly 100%, indicating excellent sampling depth across all samples (Figure 5e). The Shannon and Simpson indices ranged from 2.332 to 2.672 and 2.032 to 2.252, respectively, while the average Chao1 and ACE indices for the *B. fragilis* (*BF109* and *BF108*) groups varied from 4705 to 4403.

**Figure 6:**
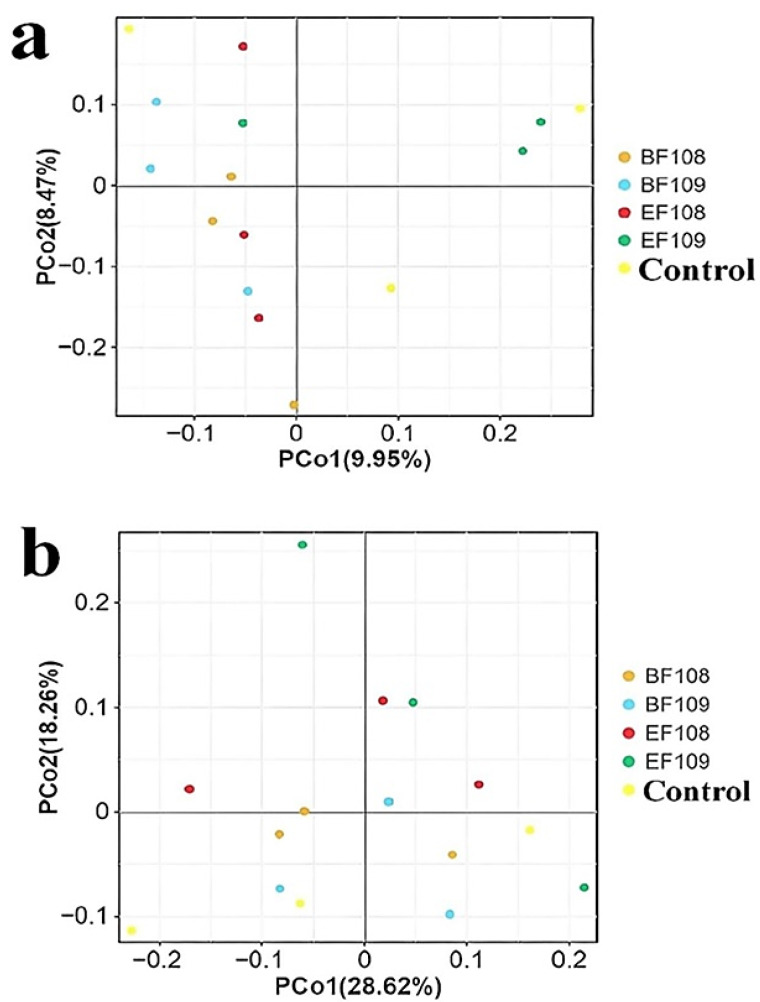
Principal coordinate analysis (PCoA) of gut microbiota across different groups. Panels (**a**,**b**) illustrate the PCoA maps based on unweighted and weighted UniFrac distances, respectively. Each colored point represents a single sample, and the differences among the groups can be assessed by the distances between these points.

The alpha diversity analyses showed no significant differences between the probiotic-treated groups and controls, indicating that the overall richness and diversity of the microbial community were not disrupted. Similarly, beta diversity analysis (PCoA) showed that samples from all groups clustered closely, suggesting no major shift in the global community structure. Despite this global stability, LEfSe analysis revealed that the probiotic intervention induced targeted, taxon-level shifts, consistent with selective modulation rather than wholesale community restructuring (Figure 6a,b).

### 3.5. Alteration the Composition of Gut-Microbiota in Different Group

**Figure 7:**
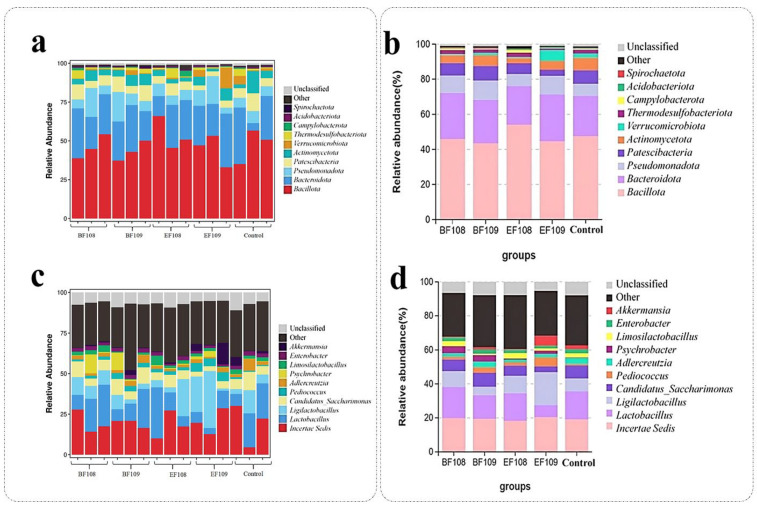
Relative abundance of the top 10 most prevalent gut microbial taxa at both phylum and genus levels among the five groups. Panels (**a**,**b**) depict the relative abundance of gut microbiota in each sample at the phylum and genus levels, respectively. Panels (**c**,**d**) show the relative abundance of gut microbiota based on the average number of each subfamily at the phylum and genus levels.

Microbial taxonomic assignment was used to determine the percentage of dominating phyla and genera in the control, *EF109*, *EF108*, *BF109*, and *BF108* groups. In all five mouse groups, *Bacillota* (47.48%, 44.4, 54.10%, 43.43%, and 45.97%), *Bacteroidota* (23.10%, 26.82%, 21.98%, 24.76%, and 26.13%), were the most prevalent phyla, accounting for approximately 97% of the taxonomic groups found (Figure 7a,b). Other phyla with smaller abundances included *Pseudomonadota*, *Patescibacteria*, *Actinomycetota Acidobacteriota*, *Verrucomicrobiota*, *Campylobacterota*, *Thermodesulfobacterota*, *Spirochaetota*, unclassified bacteria and other. In all groups (Control, *EF108*, *BF109*, and *BF108)*, *Incertae Sedis* (18.95%, 20.33%, 18.25%, 19.38%, and 19.82%) and *Lactiplantibacillus* (16.65%, 16.25%, 13.96%, and 18.31%) but not 7.09% which has been shown in *EF109*, were the most common bacterium at the genus level, respectively, followed by *Ligilactobacillus* (19.40%) which only shown in *EF109* group, accounting to the total bacterial composition (Figure 7c,d).

**Figure 8:**
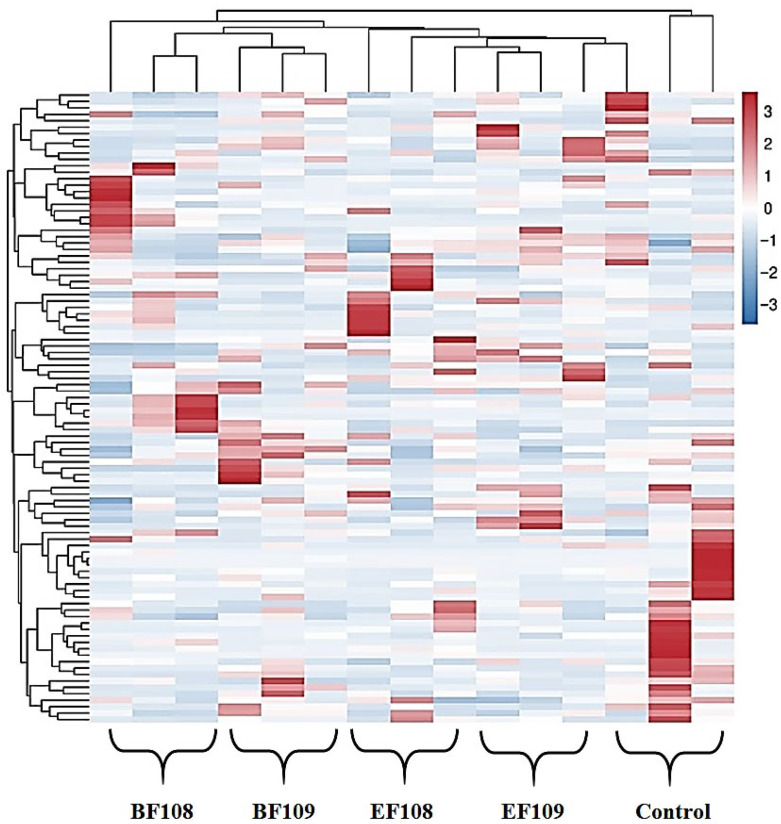
Heatmap of the relative abundance of bacterial communities at the genus level in each sample. Each colored block in the heatmap represents the relative abundance of a specific bacterial genus within that sample.

The heatmap of bacterial communities at the genus level (Figure 8) revealed distinct clustering patterns in microbial composition among the different treatment groups. Genera such as *Incertae Sedis* and *Lactobacillus* demonstrated high relative abundance across most groups, confirming their status as dominant members of the gut microbiota in this model. Notably, the probiotic candidate administration led to visible shifts in specific bacterial populations. For instance, the *EF109* group showed a unique enrichment of *Ligilactobacillus*. Furthermore, the clustering analysis indicated that the microbial profiles of the treatment groups, particularly the high-dose groups (*BF109* and *EF109*), grouped separately from the control, suggesting that the administered bacteria induced a measurable shift in the overall gut microbial community structure.

### 3.6. LEfse Analysis

**Figure 9:**
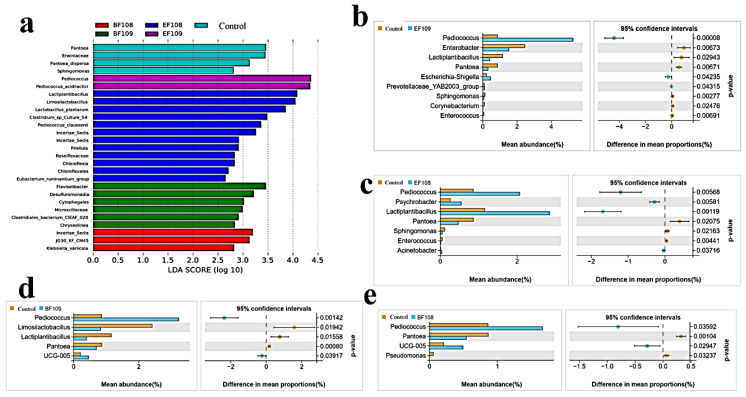
*LEfSe* analysis for the notable alterations in the differential fecal microbiota in several mouse groups. (**a**) In the five groups, the biomarker bacterial species were identified by the *LEfSe* analysis (LDA score > 4). (**b**) *t*-test analysis of the microbiota based on genus levels in groups *EF109* and control. (**c**) Variable microbiota *t*-test analysis at the genus level for Group *EF108* versus control. (**d**) *BF109* group versus control microbiota *t*-test evaluations according to genus levels, and (**e**) *t*-test comparison between control and *BF108* microbiota based on genus levels.

The differential microbiota are displayed based on *LEfSe* analysis. The findings in Figure 9a show that the control group had higher abundances of *Erwiniaceae*, *Pantoea*, and *Pantoea dispersa*. Nevertheless, the *EF109* addition group is enriched with *Pediococcus* and *Pediococcus acidilactici*, whereas the *BF109* addition group is enriched with *Microsillacaceae*, *Clostridia*, *Desulfuromonadia*, *Cytophagales*, and *Clostridiales* bacterium. Furthermore, the *Incertae Sedis* and *Eubacterium ruminantium* groups, as well as *Klebsiella variicola* and *Pediococcus clausenii*, respectively, were more prevalent in the *BF108* and *EF108* groups. *Chloroflexia* was found to be more abundant in the *EF108* group at the phylum level. The LEfse analysis results in Figure 9b–e indicated that the *EF109* addition group lowered the relative abundance of *Pantoea*, *Sphingomonas*, and *Corynebacterium*, while raising the relative abundance of *Pediococcus*, *Enterobacter*, and *Lactiplantibacillus* at the boundary level in comparison to the control group. Additionally, *Escherichia-Shigella* and *Prevotellaceae YAB2003 group* were slightly more abundant in *EF109* than in the control group. Furthermore, at the genus level, *EF108* addition increased the relative abundance of *Pediococcus*, *Lactiplantibacillus*, and *Acinetobacter*, respectively, and decreased the relative abundance of *Pantoea*, *Psychrobacter*, *Sphingomonas*, and *Enterococcus*. Compared with the control group, the *BF109* group increased the relative abundance of *Pediococcus* and decreased that of *Limosilactobacillus*, *Lactiplantibacillus*, *UCG-005*, and *Pantoea*. Meanwhile, the *BF108* group showed a lower relative abundance of *Pseudomonas*, *Pantoea*, and *UCG-005*, as well as an increase in *Pediococcus*, compared with the control group. These changes could have functional implications for gut health, such as enhanced barrier function or reduced pathogen colonization.

Notably, LEfSe analysis revealed a decreased relative abundance of the genus *Enterococcus* in the *EF108* group despite the administration of *E. faecium*. This paradoxical finding may be explained by competitive exclusion, where the administered strain outcompeted indigenous enterococci, leading to a net reduction in total genus abundance. Alternatively, our 16S rRNA methodology cannot distinguish the probiotic strain from endogenous congeners, potentially masking its successful colonization. This observation highlights the complexity of strain-level interactions and warrants future investigation using strain-specific techniques.

### 3.7. Correlation Network Analysis

**Figure 10:**
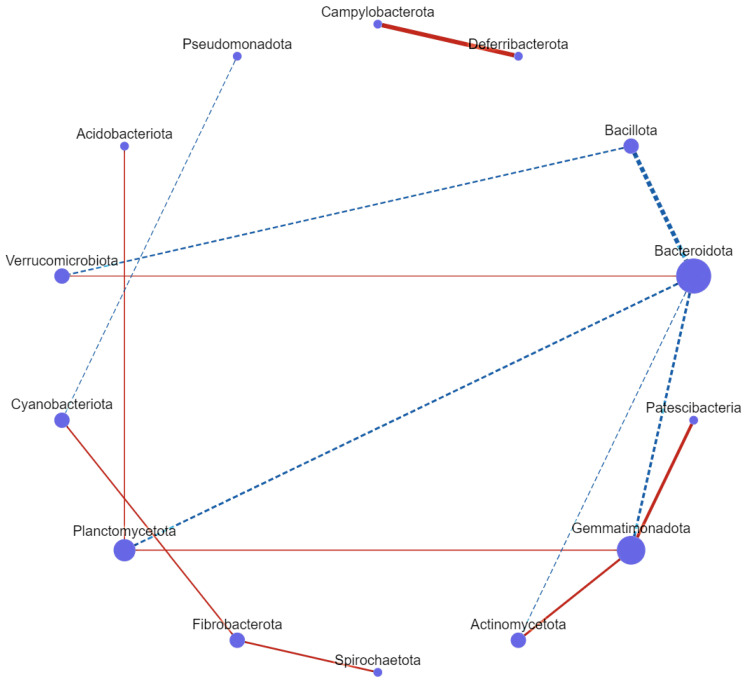
Network analysis reveals the relationship between several microbes. Red lines represent positive connections, whereas the blue lines represent a negative correlation. The control group, *EF109*, *EF108*, *BF109*, and *BF108* groups.

Correlation network analysis revealed complex microbial associations. Bacillota was positively associated with *Bacteroidota* (0.78) and *Verrucomicrobiota* (0.57). *Bacteroidota* was positively associated with *Verrucomicrobiota* (0.57) and *Planctomycetota* (0.60), and negatively associated with *Actinomycetota* (−0.51) and *Gemmatimonadota* (−0.64). Additionally, *Pseudomonadota* was negatively associated with *Cyanobacterota* (−0.51), while *Patescibacteria* and *Actinomycetota* were positively associated with *Gemmatimonadota* (0.77 and 0.67, respectively). Furthermore, *Campylobacterota* exhibited a high positive association with *Deferribacterota* (0.54). Moreover, *Spirochaetota* and *Cyanobacterota* were positively associated with *Fibrobacterota* (0.63 and 0.59), and *Acidobacterota* was positively associated with *Planctomycetota* (0.55). A notably high correlation value was also observed for *Planctomycetota* (Figure 10). These findings highlight the importance of understanding microbial interactions for developing effective probiotic interventions.

## 4. Discussion

Our hypothesis proposed that probiotic supplementation with *E. faecium* and *B. fragilis* would enhance growth metrics and modulate gut microbiota. The observed increases in body weight, reductions in liver index, and enrichment of *Pediococcus acidilactici* support this hypothesis, indicating a probiotic-driven enhancement of gut ecosystem stability. This study aimed to evaluate the potential of the probiotics *E. faecium* and *B. fragilis* in vivo, following encouraging in vitro safety evaluations. The primary markers of physiological health and the potential effects of probiotics were body weight, liver index, and colon length. The study found significant increases in body weight in the *EF108* and *BF109* groups, which may suggest improved metabolic efficiency. However, as weight gain can result from various physiological changes, not all of which are beneficial, further studies are needed to determine the exact mechanisms and health implications of this observation. However, the decrease in liver index in the *BF108* and *EF108* groups could imply reduced hepatic stress or inflammation. The lower liver index in probiotic-treated groups may reflect proportional changes due to increased body weight rather than absolute hepatic atrophy. This aligns with studies showing probiotics reduce hepatic inflammation and oxidative stress, enhancing metabolic efficiency without compromising organ integrity.

Although we acknowledge the limitation that individual feed intake was not recorded, the body weight of probiotic-treated groups showed significant improvement compared to the control. Probiotic treatments are more likely to be responsible for these weight differences than dietary variation because all groups were given the same chow diet freely, and no discernible differences in feed intake were observed.

These findings warrant further exploration to elucidate the systemic effects of these probiotics. Previous research has documented similar outcomes, indicating that probiotics can enhance food consumption and gut microbiota, thus improving growth performance in animal models [34,35]. It is noteworthy that the *BF108* and *EF108* groups exhibited significantly lower liver indices, which are often utilized to assess organ health and systemic effects. Research highlighting the role of probiotics in maintaining liver health and reducing oxidative stress suggests that a lower liver index may indicate diminished hepatic stress or inflammation [36]. This implies that, alongside promoting gut health, certain probiotic strains can offer systemic benefits.

Furthermore, segment-specific improvements in intestinal health were observed. The significant increase in colon length in the *EF109* and *BF109* groups is a recognized macroscopic indicator of improved large intestine health and reduced inflammatory status [37]. Separately, in the small intestine, histomorphological analysis revealed enhanced barrier integrity and absorptive capacity, as evidenced by increased villus height in these same high-dose groups. Collectively, our results indicate that *E. faecium* and *B. fragilis* are safe for in vivo use and may positively influence gut integrity, growth, and organ health. Future research should investigate the mechanisms that underpin these probiotic effects and assess their long-term safety and efficacy across various animal models. Deeper crypts indicate greater regenerative potential, and improved nutrition absorption typically correlates with increased villus height. According to the current research, *BF109* and *EF109* increase intestinal absorptive surface by raising villus height, while *BF108* and *EF108* primarily increase the VH/CD ratio. Additionally, the decrease in inflammatory ratings across all probiotic groups suggests that intestinal inflammation was reduced by supplementation, which could lead to better gut health.

Probiotic microbes can affect host health through diverse pathways, which can be broadly categorized into two classes: (i) interaction with the gut’s microbial ecology [38,39], and (ii) promotion of the host’s intestinal mucosal homeostasis, particularly regarding immunomodulation [40,41,42,43] and preservation of barrier function [44,45]. Using a mouse model, we examined these characteristics in vivo. It is widely accepted that a probiotic’s capacity to alter the microbial ecology of the gut is a primary driver of its potential to influence host health [9]. Thus, the initial aim of this investigation was to elucidate how treatment with the two probiotic strains, at varying dilutions, impacted the composition of the microbiota in various intestinal regions of mice. Each bacterial strain examined influenced the microbiota of the intestinal areas, impacting both bacterial load and composition. Notably, compared to mice gavaged with phosphate-buffered saline, those treated with *E. faecium* 109 and 108, as well as *B fragilis* 109 and 108, exhibited a significantly lower bacterial cell count in the intestine. In agreement with [46], a meta-analysis of 18 clinical trials suggests that probiotic supplements may be beneficial in alleviating gastrointestinal issues by reducing H2 production and mitigating gastrointestinal discomfort.

This study evaluated how *E. faecium* and *B. fragilis*, isolated from sheep, influenced the gut microbiota without detriment to the microbial community. Our findings revealed that administration of the bacterial strains diminished the presence of pathogenic organisms within the intestinal microbial community. Previous research indicates that mammalian gut microbiota evolves dynamically during development and stabilize at maturity [47]. Young animals often experience diarrheal illnesses, which may be directly linked to their maturing gut microbiota [48]. Therefore, probiotic treatment during the juvenile stage of an animal may reduce pathogenic organisms by improving the structural integrity of the intestinal microbial community [49].

The clustering of the probiotic-treated groups suggests that supplementation with *E. faecium* and *B. fragilis* contributes to the stability of the gut microbiota, potentially conferring resilience against dietary or environmental disturbances. This stabilizing effect aligns with other studies that demonstrate probiotics can prevent dysbiosis and bolster gut homeostasis [50]. Based on the alpha diversity analysis and OTU-annotated results, the colons and ceca of Hainan black goats were found to be more diverse and richer than the ileum, jejunum, and duodenum, which contrasts with previous research on the digestive contents of Yimeng black goats [51]. These discrepancies may be attributed to breed differences and feeding practices, paralleling our findings regarding OTUs and alpha diversity analysis. While the *BF108* strain may aid in decreasing dysbiosis, the *EF108* strain appears particularly effective in enhancing gut microbial diversity and richness. Future research should delve into the unique microbial taxa influenced by these probiotic strains and their functional implications for gut health.

Contrary to causing broad dysbiosis, supplementation with *E. faecium* and *B. fragilis* contributed to the stability of the overall gut microbiota structure, as evidenced by the non-significant differences in alpha and beta diversity. Importantly, this structural stability occurred alongside targeted alterations in specific microbial populations, as clearly identified by LEfSe analysis. This suggests that these probiotic candidates exert a refined, taxon-specific influence rather than a wholesale restructuring of the gut ecosystem The enrichment of recognized beneficial genera, such as *Pediococcus* and *Lactiplantibacillus*, within a stable community framework underscores their potential to enhance gut health without destabilizing the resident microbial network [52]. These results align with earlier findings showing that probiotics can stabilise gut microbial populations [53]. The impact of *E. faecium* and *B. fragilis* on the composition of the gut microbiota influences diversity indicators. By regulating the architecture of the microbial community, probiotics enhance gut health. However, the functional implications of microbial shifts, host–microbiota interactions, and immune responses require further investigation.

Moreover, previous studies indicate that gut microbial dysbiosis can influence intestinal permeability and human immunity, rendering individuals more susceptible to infections. We hypothesize that the heightened mortality and weight loss observed in diarrhoea-afflicted yaks may be connected to this condition. In the intestines of a healthy yak calf, this study found that *Firmicutes* and *Bacteroidetes* were the most prevalent bacterial phyla [54,55,56]. Consistent with our research, the predominance of *Bacillota*, *Bacteroidota*, and *Pseudomonadota* across all groups underscores their ecological and functional significance within ruminant gastrointestinal tracts. The microbial composition in the gut plays a critical role in maintaining homeostasis, with *Pathobacteria* exhibiting a unique function in host health and gut functionality. Studies suggest that *Bacteroidota* can enhance nutrient intake, while Bacillota can decompose cellulose, proteins, and carbohydrates [57]. Conversely, Bacteroidetes primarily facilitate the digestion of proteins and carbohydrates and support the development of the host’s immune system [58]. *Pseudomonadota* are crucial for sustaining the structural equilibrium and stability of the intestinal flora and serve as key indicators of an animal’s gut health [59].

At the genus level, results have demonstrated that the dominant groups within the mice’s gut comprised *Incertae sedis*, *Lactobacillus*, *Ligilactobacillus*, *Enterobacter*, *Limosilactobacillus*, *Psychrobacter*, *Adlereutzia*, and *Pediococcus*. Past studies suggest that *Lactobacillus* can enhance intestinal mucosal immunity and interact with intestinal epithelial cells to defend against entero-invasive *E. coli* [60,61]. Given that these genera are frequently linked to gut health, they may contribute to improved immune regulation and gut barrier integrity. The increase in *Enterobacter* and *Lactiplantibacillus* observed in this group is particularly noteworthy [62]. Further investigations have demonstrated that administering *Lactobacillus* to obese mice daily is beneficial in preventing non-alcoholic fatty liver disease by improving the intestinal environment and reducing inflammation [63,64]. The supplementation of *Lactobacillus* not only enhances intestinal antioxidant capacity and digestive enzyme activity but also boosts immunity and regulates gut microbiota, thereby benefiting the host [64,65]. *Sphingomonas*, known for its capacity to metabolise a range of organic materials, harbours numerous potential applications for both industrial use and environmental conservation [66]. The observed variations in *Akkermansia* abundance between the *EF109* and control groups indicate treatment-specific changes in microbial populations associated with mucin degradation.

The heat map of clustering analysis, which illustrated diverse microbial profiles across treatments, strongly corroborated these findings. While our study clarifies significant variations in microbial composition, the functional consequences of these changes remain poorly understood. Future research may employ metagenomic techniques. Reference [67] reported on the two dominant bacteria, *Prevotella* and *Ruminococcus*, in the rumen of adult ruminants, which are essential for the breakdown of cellulose and polysaccharides. The control group exhibited a higher relative abundance of *Erwiniaceae*, *Pantoea*, and *Pantoea dispersa* than the treatment groups, indicating that the lack of therapeutic intervention may have permitted less advantageous bacteria to thrive. *Pediococcus*, particularly *Pediococcus acidilactici*, is a well-known lactic acid bacterium frequently utilized in probiotic applications. This supports earlier research indicating that *Pantoea* is a typical part of gut microbiota that can become deleterious in dysbiotic contexts [68]. While the *BF109* and *BF108* treatments elevated the levels of *Clostridium* and *Microsyllaceae*, suggesting potential advantages for gut health and disease prevention, the *EF109* and *EF108* treatments encouraged the establishment of probiotic species. This study suggests that probiotic strains may enhance gut health by lowering gut pH, increasing lactic acid production, and inhibiting pathogenic microbes.

This investigation unveiled a complex network of microbial relationships, revealing substantial positive and negative correlations among various phyla. These intricate dynamics may yield valuable insights into the interactions present within microbial communities, illuminating the underlying ecological connections that influence the overall microbial equilibrium.

Positive correlations, such as those between *Gemmatiomonadota* and *Patescibacteria*, suggest a synergistic relationship, which may indicate shared environmental preferences or metabolic pathways that enhance their cooperative functions. Conversely, negative correlations involving *Pseudomonadota* and *Cyanobacteria* suggest competitive exclusion, which can disrupt community dynamics.

The possible negative impact of probiotics on weight gain may be influenced by these interactions. The introduction of probiotics can modify the pre-existing microbial community structure, resulting in changes to metabolism and nutrient absorption that do not necessarily lead to increased weight. Environmental factors may also play a role in driving these relationships; for instance, the interaction between *Campylobacterota* and *Deferribacterota* suggests a functional synergy that could support metabolic pathways and nutrient cycling, particularly in the context of degrading complex substrates. Such changes could affect overall nutrient availability and absorption, consequently influencing weight gain.

These findings support prior research on microbial interactions. For example, a study by [69] identified beneficial interactions between *Planctomycetota* and *Acidobacterota*, alongside comparable antagonistic correlations between *Bacillota* and *Bacteroidota*. By detailing a wider array of microbial interactions and providing stronger evidence for specific inter-phylum linkages, our study expands upon these findings and highlights the complex relationships within microbial ecology and their implications for ecosystem functionality.

The outcomes of this investigation demonstrated that supplementation with *E. faecium* and *B. fragilis* increased beneficial bacteria while decreasing harmful ones. Moreover, the *B. fragilis* and *E. faecium* successfully improved the microbial community structure in the mice, lowering the proportion of detrimental bacteria.

Current research confirms the safety of isolates from sheep, as no infection or tissue damage was observed in treated mice [27]. However, despite prior findings indicating survival of gastrointestinal disorders without pathological changes, comprehensive genomic-level safety characterization including whole-genome sequencing, antibiotic resistance, and virulence factor profiling has yet to be conducted. Further genetic analysis is necessary to evaluate their suitability for translational or commercial purposes.

It should be noted that the beneficial effects observed in this study may be strain-specific and context-dependent. Probiotic properties, including modulation of gut microbiota and physiological outcomes, often vary across bacterial strains even within the same species [9,70]. Moreover, some of the observed changes may represent transient responses to short-term supplementation rather than permanent alterations in host physiology. Therefore, while our findings support the potential of the tested strains to improve intestinal health, broader claims regarding probiotics in general should be made with caution, and further studies with longer follow-up periods and additional strains are warranted.

The translational potential of these findings is supported by a growing body of research in other livestock species, such as pigs, where targeted dietary interventions have demonstrated a significant capacity to modulate the gut ecosystem and improve intestinal health, underscoring the broader applicability of microbial management in animal husbandry [71,72,73].

Although the potential benefits of probiotics for humans have been extensively researched, the current study was carried out in a mouse model; therefore, it is important to use caution when extrapolating the findings to human health. Though confirmation in human trials is necessary before more general health claims can be made, our findings offer important insight into the strain-specific benefits of probiotics in mice. It will take further clinical studies to confirm whether these findings apply to human populations.

The current study’s lack of evaluation of systemic or mucosal cytokine profiles is one of its limitations. Although our histopathological data (specifically, the significantly lower inflammation scores in treated groups) indicate reduced intestinal inflammation, the specific immunomodulatory pathways involved were not elucidated. Although the sample size for 16S rRNA sequencing (n = 3 per group) was appropriate for an initial screening, future work will utilize larger cohort sizes to robustly validate these microbial shifts and enable more powerful statistical modeling of host–microbiota interactions. Additionally, the 16S rRNA sequencing used for microbial analysis, while informative, does not capture the entire microbial diversity, as evidenced by the presence of ‘unclassified’ and ‘other’ taxa, leaving a portion of the community unidentifiable at a finer resolution. Therefore, future research incorporating cytokine analysis (e.g., IL-6, IL-10, TNF-α, and IFN-γ) is warranted to provide a deeper mechanistic understanding of the anti-inflammatory effects suggested by our findings.

## 5. Conclusions

This study evaluated the safety and preliminary efficacy of *E. faecium* and *B. fragilis* isolates in a murine model. The findings demonstrate that oral administration was well-tolerated, indicating a favourable safety profile. Supplementation, particularly with strains *EF108* and *BF109*, was associated with significant increases in final body weight and colon length, alongside a reduced liver index, suggesting a potential positive influence on growth performance and intestinal morphology. Analysis of the gut microbiota revealed that probiotic administration altered microbial community structure, notably increasing the relative abundance of recognised beneficial species such as *Pediococcus acidilactici* and *Lactiplantibacillus plantarum*. Furthermore, correlation analyses indicated putative synergistic relationships between the dominant phyla *Bacillota* and *Bacteroidota* and other beneficial taxa. Collectively, these results indicate that *E. faecium* and *B. fragilis* possess attributes consistent with potential probiotic functionality. However, these findings are preliminary and derived from a controlled, short-term preclinical model. Consequently, further investigation is necessary to elucidate the mechanistic pathways involved, validate these outcomes in long-term studies and target species, and ultimately assess their practical applicability in animal production systems.

## Figures and Tables

**Table 1:** Dose of probiotic treatments administered to mice.

Group	Treatment Strain	Dose Volume (µL)	Concentration (CFU/mL)	Total CFU Per Mouse Per Day	Average Mouse Weight (g)	Equivalent CFU/kg BW/day
**Control**	PBS	200	--	--	22	--
***BF109***	High dose (*Bacteroides fragilis*)	200	1 × 10^9^	2 × 10^8^ CFU	22	9.1 × 10^9^
***EF109***	High dose (*Enterococcus faecium*)	200	1 × 10^9^	2 × 10^8^ CFU	22	9.1 × 10^9^
***BF108***	Low dose (*Bacteroides fragilis*)	100	1 × 10^8^	1 × 10^7^ CFU	22	4.5 × 10^8^
***EF108***	Low dose (*Enterococcus faecium*)	100	1 × 10^8^	1 × 10^7^ CFU	22	4.5 × 10^8^

**Table 2:** Histopathological measurements of intestinal tissues.

Group	Villus Height (µm)	Crypt Depth (µm)	VH/CD Ratio	Inflammation Score (0–1)
Control	437 ±17 ^b^	129± 3.0 ^c^	4.5 ±0.2 ^b^	0.5 ± 0.2 ^a^
*BF109*	579 ± 20 ^a^	221± 2.1 ^a^	3.4 ±0.3 ^c^	0.3 ± 0.1 ^b^
*EF109*	579 ± 20 ^a^	203 ±5.2 ^b^	4.7 ± 0.2 ^b^	0.4 ± 0.2 ^a^
*BF108*	368 ± 19 ^c^	207 ±5.0 ^b^	5.5 ± 0.1 ^a^	0.2 ± 0.1 ^c^
*EF108*	368 ± 17 ^c^	203 ± 5.1 ^b^	4.7 ± 0.3 ^b^	0.3 ± 0.2 ^b^

Values are mean ± SD (n = 3). Different superscript letters (^a^, ^b^, ^c^) indicate significant differences (*p* < 0.05).

## Data Availability

The original contributions presented in this study are included in the article. Further inquiries can be directed to the corresponding author.
